# Curcumin encapsulation and protection based on lysozyme nanoparticles

**DOI:** 10.1002/fsn3.1129

**Published:** 2019-07-17

**Authors:** Jilai Cui, Jie Zhou, Lu Huang, Junxiang Jing, Ningze Wang, Luyuan Wang

**Affiliations:** ^1^ College of Life Science Xinyang Normal University Xinyang China; ^2^ Tea Plant Biology Key Laboratory of Henan Province Xinyang China; ^3^ Institute for Conservation and Utilization of Agro‐bioresources in Dabie Mountains Xinyang China

**Keywords:** curcumin, lysozyme, nanoparticles, protection, solubilization

## Abstract

Curcumin possesses antioxidant, anti‐inflammatory, and other properties. However, this compound exhibits low bioavailability because of its poor solubility and stability. In this paper, lysozyme nanoparticles were fabricated through solvent evaporation, and then, the solubilization and protection capability of curcumin were investigated. Lysozyme nanoparticles were characterized by dynamic light scattering technique, atomic force microscopy, transmission electron microscopy, and Fourier transform infrared spectroscopy. The load capacity and stability in thermal environment were further explored. Results showed that the lysozyme nanoparticle displayed a spherical structure (127.9 ± 2.12 nm) with favorable distribution. The solubility of curcumin can increase to 22 μg/mL. After encapsulation by lysozyme nanoparticles, the retentive curcumin can reach up to 67.9% and 30.25% at 25°C and 50°C, respectively, significantly higher than that of free curcumin. Meanwhile, experiments on DPPH free radicals indicated the curcumin loaded by lysozyme nanoparticle possessed higher free radical scavenging activity than that of free curcumin with same treatments. The results confirmed that lysozyme nanoparticles exhibit potential applications in solubilizing and protecting the environment‐sensitive hydrophobic functional components.

## 1 INTRODUCTION

Curcumin (Cur) is a yellow pigment from traditional medicine turmeric and is commonly used as a spice and food‐coloring agent. Cur has drawn much attention because Cur shows multiple properties, such as anti‐inflammatory, antioxidant, decreasing hematic fat, resisting sclerosis of arterial congee appearance, and antitumor (Karimian, Pirro, Johnston, Majeed, & Sahebkar, 2017; Saberi‐Karimian et al., 2018; Wang et al., 2017). However, as a functional component and effective therapeutic agent, Cur exhibits low water solubility and relatively high environmental sensitivity to temperature and ultraviolet radiation, which severely limit its application (Nimiya et al., 2016; Zhao, Pan, Nitin, & Tikekar, 2014). Various systems, including emulsion, polysaccharide/protein complex, and nanogel, have been explored and applied to improve the stability and bioavailability of Cur (Mangalathillam et al., 2011; Tapal & Tiku, 2012; Zou et al., 2016).

In recent years, nanoparticles are commonly used in loading, protection, and delivery systems for functional components, such as polyphenols, carotenoids, vitamins, fatty acids, sterols, and other active ingredients (Chen et al., 2013). Nanoparticles improve the solubility and stability of active ingredients and expend the application fields of functional components, especially for the compounds being sensitive to the environment, such as Cur and folic acid (Das, Kasoju, & Bora, 2010; Sneharani, Karakkat, Singh, & Rao, 2010). A carrageenan/lysozyme soluble complex was prepared by one‐step self‐assembly and was found to be a suitable vehicle for solubilizing and protecting curcumin in heating and ultraviolet radiation environment (Xu et al., 2014).

In this paper, lysozyme nanoparticles (Ly NPs) were prepared by desolvation method. Ly NPs were subsequently characterized through particle size and morphology. Therefore, the encapsulation and protection of Cur were explored, and the antioxidant activity was further verified using DPPH. This endeavor attempted to provide a simple and feasible strategy for solubilization and protection of the hydrophobic functional components.

## 2 MATERIALS AND METHODS

### 2.1 Materials

Curcumin (95.0% purity), as well as lysozyme (Ly, 14.3 kDa) from chicken egg white, was purchased from Sinopharm Chemical Reagent Co., Ltd. Other chemicals were of reagent grade and used without purification. All solutions used in the experiments were prepared using ultrapure water through a Millipore (Millipore) Milli‐Q water purification system.

### 2.2 Preparation of Ly NPs

Ly NPs were prepared by desolvation (Jahanban‐Esfahlan, Dastmalchi, & Davaran, 2016). Ly was dissolved in purified water with gentle magnetic stirring for 2 hr at room temperature at the concentration of 1.0 mg/mL. Ethanol was added to Ly solutions at the volume ratio of 15:5 with further stirring for 1 hr. After becoming turbid, the solution was incubated at 80°C for 15 min. Subsequently, Ly NPs were prepared by removing ethanol through rotary evaporation at 30°C under vacuum, and the NPs were stored at 4°C for further use.

### 2.3 Characterization of Ly NPs

The particle size and zeta potential (ζ) of Ly NPs were measured with Nano‐ZS90 at 25°C (Melvin Instruments) according to Xu (Xu, Ge, et al., 2018). The Fourier transform infrared (FT‐IR) spectra were recorded using a Nicolet Nexus 470 spectrometer with 32 scans and resolution of 4 cm^−1^ in the range of 400–4000 cm^−1^. The morphology of the complex was investigated with a JEOL transmission electron microscope (TEM) (H‐7650, Hitachi). During the TEM test, a drop of Ly NPs solution was dispensed directly onto a carbon‐coated copper grid and allowed to dry spontaneously.

Morphology of the particles was determined using atomic force microscopy (AFM) (Wei et al., 2015). Tapping mode measurements were performed in air using a Dimension Icon with a Nanoscope IV controller (Bruker Corporation) and silicon cantilevers with tetrahedral tips (OMCL‐AC160TS—Olympus) with a nominative force constant of 42 Nm^−1^ and a resonance frequency of around 300 kHz. For sample preparation, undiluted nanosuspensions were dropped onto a freshly cleaved mica surface (Plano GmbH). After 5 min, the liquid was removed with a tissue and the samples were completely dried before imaging.

### 2.4 Cur encapsulation based on Ly NPs

**Figure 1:**
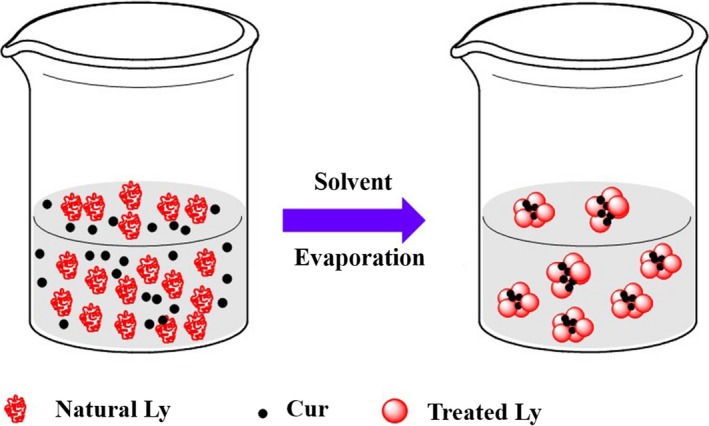
Tentative encapsulation scheme of Cur based on Ly NPs

Cur was firstly dissolved in ethanol and then added into the Ly NPs solution with different final concentrations (5, 8, 10, 13 mg/mL) for encapsulation according to previous reports as described in Figure 1 (Naksuriya et al., 2015). Cur concentration was determined using a UV‐vis spectrophotometer (UV‐1100, MAPADA) at 428 nm (Moussa, Hmadeh, Abiad, Dib, & Patra, 2016; Nazari‐Vanani, Moezi, & Heli, 2017). Free Cur (the supernatant solution) solution was obtained after centrifugation at 1888 *g* for 30 min at 4°C, and the content of Cur was estimated from a standard *X‐Y* plot of Cur (dissolved in ethanol) versus concentration. Encapsulation efficiency (EE) and loading capacity (LC) were defined as the drug content encapsulated into Ly NPs and calculated as follows (Xu et al., 2019):

$$\text{EE}\left(\%\right)=\frac{\text{Total}\;\text{Cur}-\text{Free}\;\text{Cur}}{\text{Total}\;\text{Cur}}\times 100$$

$$\text{LC}\;\left(\mu g/g\right)=\frac{\text{Total}\;\text{Cur}-\text{Free}\;\text{Cur}}{\text{Weight}\;\text{of}\;\text{Ly}\;\text{NPs}}\times 100$$

### 2.5 Cur protection by Ly NPs

To evaluate the effect of encapsulation on the stability of Cur against heat treatment, Cur retention rate was comparatively studied. Free Cur solution and Cur‐loaded NPs solution at 10 μg/mL (in water) shared 25°C and 50°C treatment for 30 min. Free Cur was prepared equally to the encapsulated Cur in which water was replaced by the Ly NPs solution. For the Cur‐encapsulated Ly NPs, ethanol was added equally, stirred for 4 hr, and then evaporated overnight at 40°C under vacuum (Kumar & Ahuja, 2013). The retention rate of Cur was calculated through the ratio of absorbance at 428 nm.

### 2.6 Antioxidant activity of free and encapsulated Cur

Antioxidant activity was further verified using DPPH method after heat treatment. In brief, free Cur solution and Cur‐loaded NPs solution at 10 μg/mL (in ethanol) were suffered at 25ºC and 50ºC for 30 min. The scavenging activity assay was carried out by recording the absorbance of DPPH solution (100 μM) at 517 nm in the presence and absence of the remnant Cur with a UV‐vis spectrophotometer (Hamlaoui, Bencheraiet, Bensegueni, & Bencharif, 2017; Tonelli et al., 2019). The free radical scavenging potency of the curcumin was expressed as the percentage of DPPH that was decreased compared with that of the control condition after 30 min of preservation in the dark.

## 3 RESULTS AND DISCUSSION

**Figure 2:**
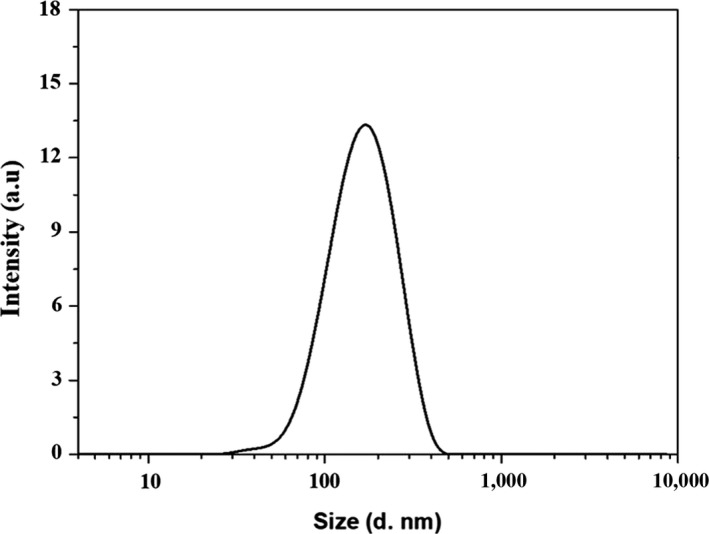
Size distribution of Ly NPs

**Figure 3:**
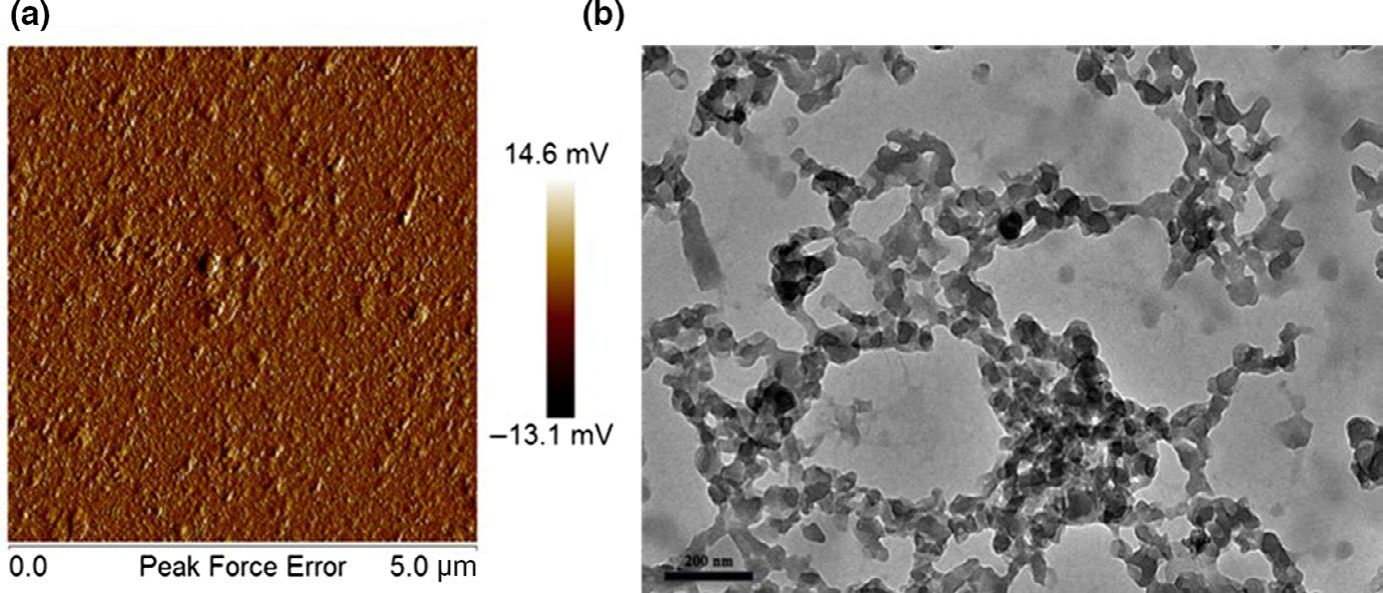
AFM (a) and TEM (b) microstructure of Ly NPs

Figure 2 shows that the average particle size of the prepared Ly NPs was 127.9 ± 2.12 nm. The size distribution index (PDI) of Ly NPs was 0.29 ± 0.03. The microscopic morphology of Ly NPs was observed through atomic force microscopy (AFM) and TEM (Figure 3). AFM and TEM results showed that Ly NPs displayed elliptical appearance with a diameter probably around 90 nm and good dispersivity. The measured particle size was smaller than the hydrate particle size.

**Figure 4:**
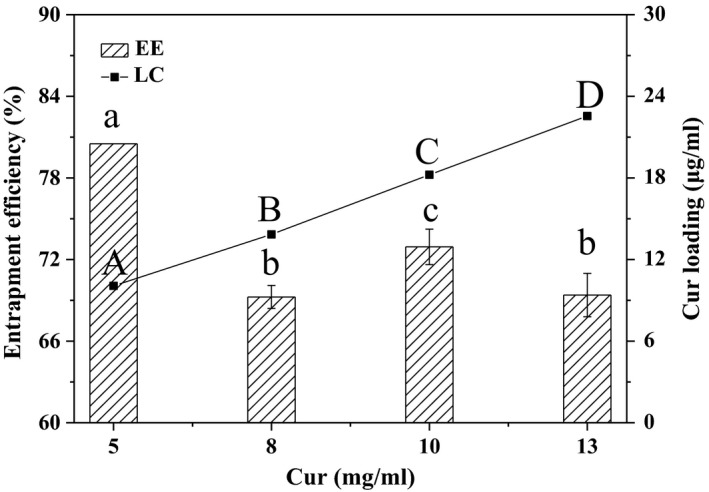
EE and LC of Cur based on Ly NPs. EE change was shown as bar chart (left axis), and LC change was shown as line chart. Different letters in lowercase mean statistical differences in Entrapment efficiency (EE), and different letters in upper case mean statistical difference in curcumin loading (LC)

Cur is recognized as a physiological and pharmacological functional nutritional component. Figure 4 illustrates that the hydrophobic micro area of Ly NPs can improve the solubility of Cur. The EEs of Cur were different at different concentrations. At the concentration of 5 mg/mL, EE reached 80.5%. The EE then decreased with increasing Cur concentration, indicating lower loading capacity at lower Cur concentration. By contrast, LC continued to increase with higher concentration of Cur, and LC reached 22.55 ± 0.08 μg/mg at the Cur concentration of 13 ± 0.02 mg/mL. When the concentration of Cur was 10 mg/mL, EE and LC were 72.93%±1.3% and 18.23 ± 0.05 μg/mg, respectively.

**Figure 5:**
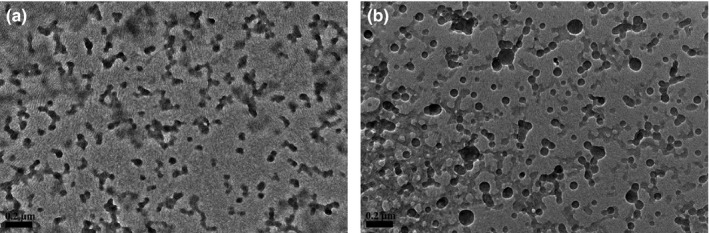
TEM of free Ly NPs (a) and Cur‐loaded Ly NPs (b). Scale bar = 0.2 μm

**Figure 6:**
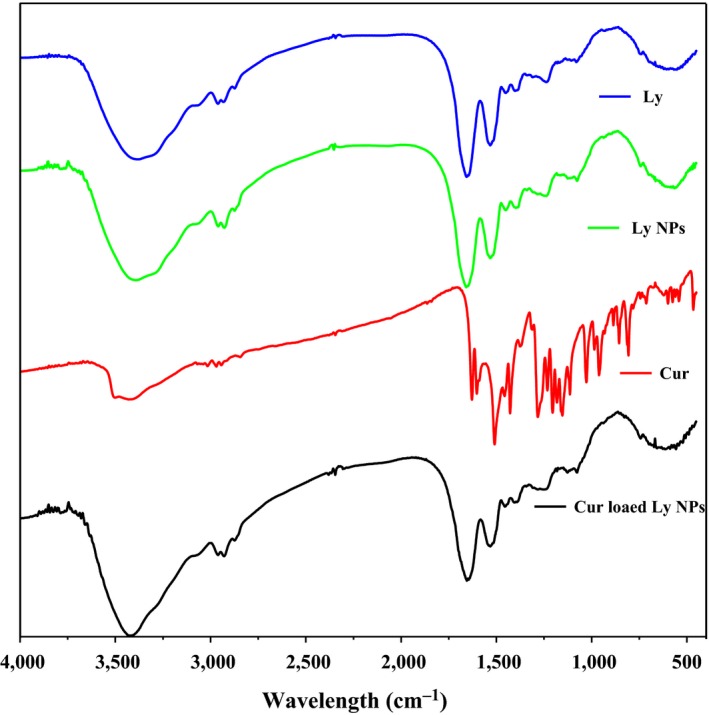
FT‐IR spectra of Ly, Cur, Ly NPs, and Cur‐loaded Ly NPs, respectively, from top to bottom

For exploring the influence of Cur loading on the structure of Ly NPs, the microscopic appearance was visualized through TEM after Cur encapsulation (Figure 5). The result showed that the Cur packaged Ly NPs displayed better dispersion. FT‐IR spectroscopy was used to explore the change of molecular group during Ly NPs fabrication and the interaction between Ly and Cur (Figure 6). The FT‐IR spectra of Ly NPs showed coincident absorption peaks with those of Ly, including the characteristic absorption peaks of amide I, amide II, and amide II. The significant change observed around the 3,500 cm^−1^ range, possibly due to N‐H or O‐H stretches. However, the absorption peak of Ly NPs shifted compared with that of Ly. The phenomenon was resulted from the partly denaturation during NPs preparation. It also found in the other protein during NPs fabrication through heat treatment (Liufeng et al., 2015; Xu, Jin, et al., 2018). After encapsulation of the Cur‐loaded Ly NPs did not show new absorption peak compared with Cur and Ly NPs.

**Figure 7:**
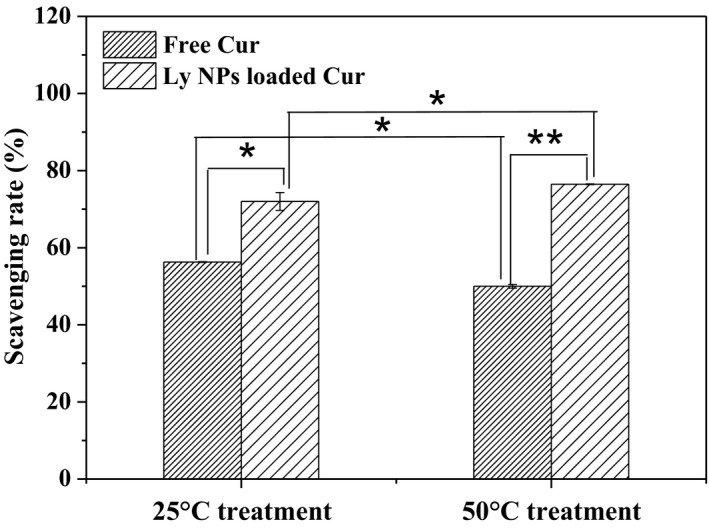
Radical scavenging activity improvements by Cur entrapment. One asterisk means statistically *p* < 0.05, and two asterisks mean very significant difference with *p* < 0.01

For free Cur, the degradation behavior with different rates occurred at 25°C and 50°C. The survival rates were 58.6% and 15.4% after 30 min of treatment. The heat‐induced Cur degradation behavior has been confirmed by other reports (Ko, Chang, Wang, Wang, & Hsieh, 2015; Refat, 2013). Figure 7 shows that in all cases the antioxidant activity of Cur decreased after heat treatment, indicating its sensibility in thermal environment (Paramera, Konteles, & Karathanos, 2011). The free Cur exhibited high radical scavenging activity at 56.3% and decreased to 50% after heat treatment at 50°C for 30 min. The DPPH radical scavenging activity of packaged Cur increased to 71.9% and 76.5% after treatment at 25°C and 50°C for 30 min, respectively.

## 4 DISCUSSION

Ly NPs displayed a single favorable peak distribution with the PDI less than 0.3 and good dispersivity of the prepared Ly NPs (Zhu et al., 2013). The size distribution of Ly NPs was resulted from swelling behavior in the water environment and dehydration during drying. The size of Ly NPs became smaller as measured by TEM. This phenomenon was also widely observed in other similar studies (Lin et al., 2015).

Cur offers potential applications in the fields of functional food and medicine, and has attracted considerable attention and research in recent years. However, its low water solubility and stability result in low bioavailability, which notably limits its wide application. Entrapment of Cur based on Ly NPs indicated that 72% (7.3 ± 0.13 μg/mL, 5.07 × 10^−5^ mol/L) of Cur was loaded in Ly NPs. Solubility improved by approximately 664 times compared with that in the water (11 ng/mL) (Kaminaga et al., 2003). The improvement in Cur water solubility was an order of magnitude higher than those in previous reports based on beta‐casein micelles and hydrophobic modified starch (7.5 × 10^−5^ mol/L, 1 × 10^−5^ mol/L) (Letchford & Burt, 2007; Yu & Huang, 2010). This phenomenon may be induced by the exposure of the hydrophobic group in the hydrophobic cavity of Ly during self‐assembly. This result illustrated that Ly NPs exhibits efficient encapsulation ability of Cur and potential for loading other hydrophobic health‐beneficial compounds. After entrapment, the Cur loading endowed high steric hindrance effect between Ly NPs. Ly NPs were difficult to aggregate and exhibited improved elliptic morphology. FT‐IR further illustrated that the structure of Ly was partially damaged. These results indicated that Cur was loaded driven by thermodynamics and self‐assembled by physical interaction. This phenomenon was also confirmed by Cur‐encapsulated zein nanoparticle, which indicates that hydrophobic interaction was the main force between the Cur and Ly NPs (Liang et al., 2015).

**Figure 8:**
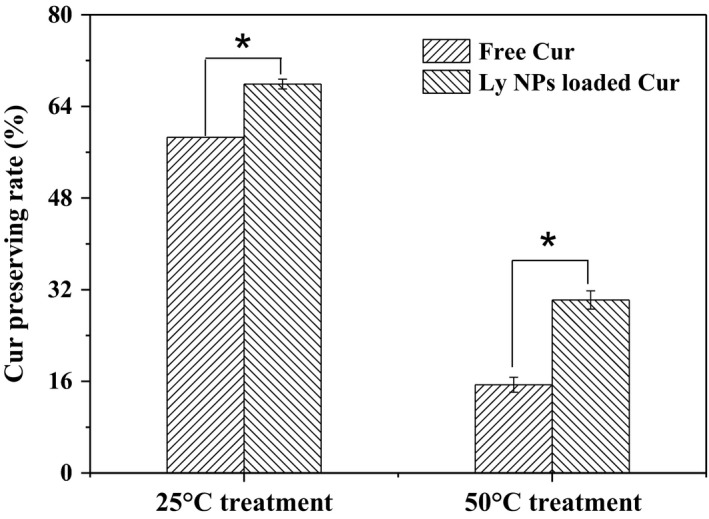
Cur protections by Ly NPs in the heat treatment. Cur preserving rates are significant different (*p* < 0.05) for free and encapsulated Cur in both 25°C and 50°C treatments

Temperature and other factors affect the structure and activity of Cur. However, heating is a conventional technique in food processing, especially for sterilization. Figure 8 shows that the Ly NPs package strategy is a simple and effective method to alleviate the degradation of Cur against thermal treatment. Obviously, compared with free Cur, the stability of Cur was significantly improved through encapsulation into Ly NPs. Interestingly, high processing temperature brought high Cur rate. Comparatively, the Cur retention rate increased to 67.9% and 30.25% after Ly NPs package with the same treatment. Therefore, the strategy of Ly NPs package not only solubilized Cur but also alleviated the degradation behavior in the adverse environments. The attempt may expand the application of Cur in food field, especially in the application of high temperature rapid processing products. The antioxidant capacity improved by 20% for the packaged Cur at 50°C. The DPPH experiment verified that encapsulating Cur in Ly NPs alleviated its breakdown during food heating process and protected its antioxidant activity.

In conclusion, Ly NPs with average size of 127.9 ± 2.12 nm were prepared through desolvation method. The prepared Ly NPs exhibit regular elliptical appearance and good size distribution. During fabrication, the structure of Ly was partially damaged. Cur was loaded as driven by thermodynamics and self‐assembled by physical interaction, which resulted in improved appearance and dispersion. At the Cur concentration of 10 mg/mL, EE and LC values were 72.93 ± 1.3% and 18.23 ± 0.05 μg/mg, respectively. Water solubility of encapsulated Cur increased by approximately 660 times. Compared with free Cur, packaged Cur displayed significantly improved survival rate after heat treatment. Increased processing temperature translates to enhanced Cur survival rate. DPPH test also showed that packaged Cur exhibited high antioxidant capability after 50°C treatment. Thus, encapsulation of Cur in Ly NPs is a practical method for the encapsulation and protection of Cur, possibly providing a workable reference for other environment‐sensitive hydrophobic functional components.

## CONFLICT OF INTEREST

The authors declare that they do not have any conflict of interest.

## ETHICAL APPROVAL

This study does not involve any human or animal testing.
